# Lack of SARS Transmission among Healthcare Workers, United States

**DOI:** 10.3201/eid1002.030793

**Published:** 2004-02

**Authors:** Benjamin J. Park, Angela J. Peck, Matthew J. Kuehnert, Claire Newbern, Chad Smelser, James A. Comer, Daniel Jernigan, L. Clifford McDonald

**Affiliations:** * Centers for Disease Control and Prevention, Atlanta, Georgia, USA; † Philadelphia Department of Public Health, Philadelphia, Pennsylvania, USA; ‡ New Mexico Department of Health, Albuquerque, New Mexico, USA

**Keywords:** severe acute respiratory distress syndrome, healthcare workers, nosocomial, transmission

## Abstract

Healthcare workers accounted for a large proportion of persons with severe acute respiratory syndrome (SARS) during the worldwide epidemic of early 2003. We conducted an investigation of healthcare workers exposed to laboratory-confirmed SARS patients in the United States to evaluate infection-control practices and possible SARS-associated coronavirus (SARS-CoV) transmission. We identified 110 healthcare workers with exposure within droplet range (i.e., 3 feet) to six SARS-CoV–positive patients. Forty-five healthcare workers had exposure without any mask use, 72 had exposure without eye protection, and 40 reported direct skin-to-skin contact. Potential droplet- and aerosol-generating procedures were infrequent: 5% of healthcare workers manipulated a patient’s airway, and 4% administered aerosolized medication. Despite numerous unprotected exposures, there was no serologic evidence of healthcare-related SARS-CoV transmission. Lack of transmission in the United States may be related to the relative absence of high-risk procedures or patients, factors that may place healthcare workers at higher risk for infection.

The epidemic of severe acute respiratory syndrome (SARS) quickly spread worldwide in 2003. As of July 11, 2003, a total of 29 countries had reported 8,427 probable cases to the World Health Organization (*1*). Much of the disease worldwide was associated with hospital-based outbreaks (*2*,*3*). Healthcare workers made up a large proportion of cases; accounting for 37%–63% of suspected SARS cases in highly affected countries (*4*–*6*). In the United States, the epidemic was limited; 74 probable and 8 laboratory-confirmed case-patients were reported, despite aggressive efforts at detection, particularly in groups at high risk. Surveillance for symptoms of SARS was recommended for all healthcare workers who were exposed to patients meeting the clinical case definition for suspected or probable SARS (*7*).

Due to the importance of healthcare facilities in transmission of SARS worldwide, state and local health departments, together with the Centers for Disease Control and Prevention (CDC), conducted a review of U.S. healthcare workers exposed to patients positive for SARS-associated coronavirus (SARS-CoV). Our objectives were to characterize the types of exposures and infection-control practices that occurred in U.S. hospitals related to SARS patient care and to determine the extent of SARS-CoV transmission to U.S. healthcare workers.

## Methods

This investigation focused on healthcare workers at highest risk for infection, in other words, those who had known unprotected exposure to laboratory-confirmed SARS-CoV–positive patients. An exposure was defined as any healthcare worker–patient interaction that occurred within droplet range (i.e., 3 feet). Exposures were categorized as either unprotected or protected, depending upon whether full personal protective equipment was used. Full equipment was defined as the use of all the personal protective equipment recommended for the care of SARS patients, i.e., a full-length gown, gloves, N95 or higher respirator, and eye protection with goggles or a face shield (*8*).

Healthcare workers were identified by hospital infection-control practitioners and public health officials through informal interviews with hospital staff, by review of employee records, and by self-identification. In addition to the healthcare workers at highest risk, other healthcare workers of interest were included, such as those with multiple protected exposures and any who requested inclusion because of concerns about exposure.

This investigation was conducted as part of the public health response to the SARS outbreak. Informed consent was obtained from healthcare workers before epidemiologic and clinical information and biologic specimens were collected. A standardized questionnaire was used to collect data on demographics, occupation, exposure characteristics, use of personal protective equipment, patient events to which the healthcare workers were exposed (e.g., coughing or vomiting), and presence during medical procedures. In addition, information was collected regarding any clinical signs or symptoms in the worker up to 10 days after exposure, including fever, cough, shortness of breath, or radiographically confirmed pneumonia. A single convalescent-phase serum sample was collected from healthcare workers at least 28 days after their last exposure to the patient. In some situations early in the outbreak, samples were collected between days 22 to 28 early in the outbreak, consistent with CDC recommendations at the time. Serum samples were tested for anti–SARS-CoV serum antibodies by enzyme-linked immunosorbent assay (ELISA) and indirect fluorescent antibody test (*9*).

Data were entered into Microsoft Access and statistical analysis was performed with SAS version 8.2 (SAS Institute, Cary, NC). Univariate analysis was performed by using two-sided Fisher exact or Mantel-Haenszel chi-squared test, as appropriate. A p value of <0.05 was considered significant.

## Results

Eight of the nine United States healthcare facilities in which SARS-CoV–infected patients were evaluated participated in the investigation. Six of the eight SARS-CoV–positive patients visited or were hospitalized at these eight facilities. A total of 110 healthcare workers (range 2–36 healthcare workers per healthcare facility) participated in this follow-up investigation (Table 1). This total represented approximately 85% of healthcare workers who were identified as being at high risk for infection. Healthcare workers were exposed to these patients from March 15 to June 23, 2003.

**Table 1:** Characteristics of SARS patient healthcare in participating U.S. healthcare facilities^a^

HCF	SARS patient	Date^b^	Date full IC^c^ started	Patient-days in HCF	Participating HCWs
1	A	3/15/03	3/15/03	10	36
2	B	3/2/03	Not started	15	7
3	C	3/14/03	3/16/03	8	16
4	D	3/20/03	3/20/03	8	7
5	E	4/6/03	Not started	1	4
6	E	4/10/03	Not started	1	7
7	E	4/14/03	4/14/03	7	21
8	F	5/27/03	Not started	4	12
^a^SARS, severe acute respiratory syndrome; HCF, healthcare facility; IC, infection control; HCWs, healthcare workers. ^b^Date, refers to the first date of the visit at the healthcare facility. This may be the date of admission or the date of visit to an outpatient clinic, emergency room, laboratory, or radiology suite. ^c^Full infection control consists of negative-pressure isolation, N95 or higher respirator, gown, gloves, and eye protection.					

The median age of healthcare workers was 41 years (range 23–61), 75% were females, and 76% were Caucasian (Table 2). The most common occupation was nursing staff (48%), and the most common work site was the medical ward (38%), followed by the emergency department (24%) (Table 2). Preexisting medical conditions in the healthcare workers were infrequent (data not shown).

**Table 2:** Demographic characteristics, occupation, and location of participating HCWs exposed to laboratory-confirmed SARS patients (n = 110)^a^

Characteristic	n (%)
Median age	41 (range 23–61)
Female gender	82 (75)
Caucasian	81 (74)
Nursing staff^b^	53 (48)
Technicians^c^	23 (21)
Medical staff^d^	16 (15)
Other occupation	18 (16)
Medical ward	41 (38)
Emergency department	26 (24)
Outpatient clinic	16 (15)
Intensive care unit	7 (6)
Other location	20 (18)

aHCWs, healthcare workers; SARS, severe acute respiratory syndrome. ^b^Nursing staff, registered nurses, licensed practicing nurses, nurses aides, patient care technician. cTechnicians, respiratory therapist, phlebotomist, radiology technician. dMedical staff, residents, fellows, attending physician, physician assistants.

Each healthcare worker was exposed over a median of 2.0 days (range 1–14), during which a median of 3.0 interactions (range 1–50) with the SARS patient occurred. Of the 102 healthcare workers from whom complete data were available, 45 (44%) reported exposure without any type of mask; 72 (70%) had exposure without eye protection (Table 3).

**Table 3:** Personal protective equipment use in HCWs reporting droplet-range exposure (within 3 feet) to a laboratory-confirmed SARS patient (n = 102)^a^

Non-use of personal protective equipment	n (%)
Without any mask	45 (44)
Without N95 or higher respirator	49 (48)
Without eye protection	72 (70)
Direct contact without gloves	40 (39)

^a^HCWs, healthcare workers; SARS, severe acute respiratory syndrome.

Sixty-six healthcare workers (65%) reported that the patient was coughing during one or more patient-worker interactions. Of these, 40% had at least one exposure without a respirator and 52% had at least one without gown, gloves, and eye protection. Eleven (11%) reported interaction with a patient who had active diarrhea, and 1 (1%) reported exposure during patient vomiting (Table 4). Healthcare procedures with high potential to generate droplets and aerosols were infrequent: 5 healthcare workers (5%) reported manipulating an airway, (i.e., performing endotracheal intubation or suctioning), and 4 (4%) reported being present during administration of aerosolized medications (Table 4).

**Table 4:** Healthcare workers reporting exposure to a laboratory-confirmed SARS^a^ patient according to patient events, healthcare procedures, and concurrent use of personal protective equipment (n = 102)^a^

Procedure or patient event	Total HCWs	Without respirator (%)	Without gown, gloves, and eye protection (%)
Coughing	66	27 (40)	34 (52)
Diarrhea	11	4 (36)	6 (55)
Airway manipulation	5	NA	NA
Aerosolized medication	4	1 (25)	1 (25)
Resuscitation	1	NA	NA
Bronchoscopy	1	0 (0)	0 (0)

^a^SARS, severe acute respiratory syndrome; HCWs, healthcare workers; NA, not available due to incompleteness of reporting.

Three healthcare facilities instituted full infection-control precautions (i.e., full use of personal protective equipment and placement in an isolation room) on the first day the patient was seen. Healthcare workers in these facilities reported significantly fewer unprotected exposures, in comparison to facilities where full SARS precautions were not instituted on the first day (62% vs. 87%, p < 0.05).

To assess adherence to infection-control practices, we identified healthcare workers who had all of their exposures only after full SARS precautions were started. We identified 43 such workers, representing all of the healthcare facilities that instituted precautions. In these workers, lapses in infection control still occurred, with nearly half reporting unprotected exposures, including many with no eye protection (Table 5).

**Table 5:** Unprotected exposures in healthcare workers exposed to laboratory-confirmed SARS patients after full infection-control procedures were initiated (n = 43)^a^

Exposure type	n (%)
Any unprotected exposure	21 (49)
Without eye protection	18 (42)
Without N95 or higher respirator	6 (14)
Direct contact without gloves	6 (14)

^a^SARS, severe acute respiratory syndrome.

Clinical signs or symptoms developed in 17 healthcare workers (15%) after exposure to one of the laboratory-confirmed SARS patients, most commonly cough (Table 6). Convalescent-phase serum samples were available for 103 (94%) healthcare workers; none (0%) tested positive for SARS-CoV.

**Table 6:** Outcomes of healthcare workers who were exposed to laboratory-confirmed SARS patients, United States (n = 110)^a^

Outcome^b^	n (%)
Cough	16 (15)
Shortness of breath	3 (3)
Fever	3 (3)
Pneumonia by chest radiography	1 (1)
Hospitalized	1 (1)

^a^SARS, severe acute respiratory syndrome. ^b^Each healthcare worker may have >1 outcome.

During the outbreak, CDC recommended furlough for any exposed healthcare worker in whom symptoms developed within 10 days of last exposure. Fifteen healthcare workers in this review (14%) were excluded from all or selected duties as a result of SARS exposure. Of these, seven reported symptoms (fever, respiratory symptoms, or radiographically confirmed pneumonia), and eight were asymptomatic. However, 10 symptomatic healthcare workers were not excluded from duty, including four nurses or nurses’ aides and one physician.

## Discussion

While healthcare-related outbreaks of SARS forced hospital closings and mandatory quarantines in some countries, no such events were reported in the United States. Our investigation demonstrates that although many U.S. healthcare workers had unprotected exposures, no documented transmission of SARS-CoV was found. In light of the numerous healthcare workers in our investigation with unprotected droplet-range exposures, lack of transmission in U.S. hospitals may have resulted from a relative absence of highly infectious patients or high-risk patient procedures.

The mode of transmission of SARS is unclear, but evidence suggests it may be spread by large- and medium-sized droplets spread within 3 feet (*5*,*10*). In one case-control study, use of any mask was associated with lower odds of infection in healthcare-related clusters (*10*).

Globally, outbreaks among healthcare workers have occurred after exposure to certain patients or at certain points during illness (*3*,*10*–*12*). In Singapore, five patients were identified early in the epidemic who had infected >10 contacts each (*10*). The timing of exposure to ill patients also is critical; patients may be most infectious in the second week of illness, as some data suggest peak viral shedding occurs at day 10 (*13*). Additionally, descriptive data suggest that severely ill patients may spread virus more efficiently, particularly if they are coughing or vomiting (*12*). Although coughing was frequently reported, vomiting was infrequent. In addition, patients seen in the United States, with the exception of one patient who required intubation, were generally not very ill.

Transmission may also be event-dependent. Procedures such as intubations and medication nebulizers have been associated with healthcare-related outbreaks, even among protected healthcare workers (*11*,*12*). One such cluster occurred in Toronto, where illness consistent with suspected or probable SARS developed in nine healthcare workers who cared for a patient around the time of intubation, despite use of full personal protective equipment (*12*). In the United States, potential droplet- and aerosol-generating procedures were infrequent: only one patient required mechanical ventilation, and few healthcare workers reported administering aerosolized medication or performing bronchoscopy. One notable exception was a worker who performed two endotracheal intubations before SARS was diagnosed. However, despite wearing only an N95 mask and gloves, this healthcare worker did not become symptomatic or seroconvert.

Our study was subject to a number of limitations. First, enrollment of both healthcare facilities and healthcare workers was incomplete. One institution in which healthcare workers were exposed to two SARS-CoV–positive patients was not included. Active surveillance performed by state and local public health officials, as well as hospital infection-control practitioners, identified no symptomatic healthcare workers among the exposed (J. Rosenberg, pers. comm.). Also, completeness of recruiting varied between institutions, although we had a high participation rate overall of approximately 85% of healthcare workers identified as being at high risk.

As in all surveys, recall bias was a concern. However, given that no healthcare workers were SARS-CoV–positive and few had symptoms, the effect of outcome on recall was probably minimal. Additionally, questions about hand hygiene and removal of personal protective equipment were not included because of concerns of overwhelming bias inherent in recalling such practices, although these factors may have been important.

Third, although most serum samples were obtained >28 days after last exposure to the SARS patient, 19 (18%) samples were obtained during days 22 to 28. These samples were primarily collected early in the outbreak when the recommendation for convalescent-phase serum collection was set for >21 days after exposure. Evidence from other studies shows that most case-patients case will seroconvert by day 20 (*13*). Although this ELISA is currently used as a standard criterion and has unknown sensitivity, a similar assay has been reported to have an estimated sensitivity of approximately 93%, based on clinical case definitions for probable SARS (*13*).

Despite the limitations of the study, a number of insights were gained from this analysis that may help prepare public health officials and clinicians for a reappearance of SARS, should it occur, or for the emergence of another infectious disease. Rapid identification and isolation of potentially infectious persons undoubtedly will help minimize exposures. Communication between public health officials and hospital infection control staff can help with efficient implementation of control procedures.

However, current levels of adherence to infection-control practices in the United States may not be sufficient if many high-risk patients or procedures are encountered. Unprotected exposures among healthcare workers may still occur despite implementation of facilitywide infection-control precautions. Therefore, new initiatives for infection control should include measures to improve compliance with personal protective equipment overall, in addition to specifically focusing on patients and events that have the highest risk for transmission.
